# Nursing Education in a Real-Life Context: The Teaching Ward Round

**DOI:** 10.3390/nursrep11010005

**Published:** 2021-01-27

**Authors:** Juan Miguel Martínez-Galiano, Laura Parra-Anguita, Miguel Delgado-Rodríguez, Manuel González-Cabrera

**Affiliations:** 1 Department of Nursing, University of Jaén, 23071 Jaén, Spain; jgaliano@ujaen.es (J.M.M.-G.); mgonzale@ujaen.es (M.G.-C.); 2 Consortium for Biomedical Research in Epidemiology and Public Health (CIBERESP), 28029 Madrid, Spain; mdelgado@ujaen.es; 3 Department of Health Science, University of Jaén, 23071 Jaén, Spain; 4 Department of Emergency and Critical Care, San Agustín University Hospital, 23700 Linares, Spain

**Keywords:** innovation, nursing, nursing training, teaching methodology, teaching rounds

## Abstract

Education in nursing is continually changing. The didactic methods used in other fields may be useful for closing the gap between theoretical learning and the reality of practical nursing. This study aimed to determine the association between a teaching model centered on the reality of nursing care, which is individualized to each context, and knowledge acquisition. A controlled experimental study was conducted with random allocation to two groups of students in their second year of a nursing degree (University of Jaén). The control group undertook practical work placements according to the traditional model. The intervention group participated in a “teaching round” during their practical placements. Knowledge tests were conducted after the placements. No significant differences were found for age or education level between the students of the control group (*n* = 46) and the intervention group (*n* = 48). In terms of the association between participation in the teaching round and the knowledge test (maximum score of 10), the mean grade in the intervention group was 8.83 ± 0.22, while it was 7.68 ± 0.23 in the control group (*p* = 0.001). The teaching round increased the student’s acquisition of knowledge, even though this was not reflected in the global grade of the course.

## 1. Introduction

Nursing education has undergone several significant changes in response to the challenges faced by healthcare organizations [1]. One of the current challenges of the teaching–learning process is to design appropriate teaching methods for the acquisition of specific competencies, as well as evaluation criteria and procedures to verify whether these competencies have actually been acquired [2].

In the health sciences disciplines, there is sometimes a gap between what is taught in university and what the reality of care demands. The acquisition of quality clinical experience within a pedagogically oriented clinical learning environment is an important educational issue [3]. Future healthcare professionals must acquire, in addition to theoretical knowledge, specific skills and abilities that cannot always be gained in training through practical seminars or simulations in recreated scenarios. Therefore, in the higher education of health disciplines in which the clinical learning environment is important, practical placements in health centers are included so that students can achieve the desired learning outcomes [4].

One of the nursing degree characteristics is the requirement to develop skills and working methods more in line with the needs of the person, family, and community, along with the acquisition of skills, abilities, and aptitudes via practical teaching in healthcare centers [5]. Nursing studies in Spain are organized to include theory with classes in large groups. Another part of the studies is developed with theoretical–practical methods, in which students have to demonstrate their skill in teaching laboratories with simulators and also participate in clinical placements in health centers.

One of the methods that can be applied in these clinical placements in healthcare centers is the teaching round. The teaching round has both teaching and care objectives. For the correct application of this method, various factors are required: a professor responsible for the teaching, the participation of all involved, discipline, and organizational and resource planning [6].

The teaching round has been used for a long time in the education process in the medical field [6,7,8,9]. It was later introduced in the teaching of nurses [10]. Numerous authors have highlighted the importance of the teaching round for the training of future professionals. They emphasized the complexity of the method and its importance in the training of professionals of excellent quality. The method encourages the use of clinical judgment in decision-making, the development of the necessary professional skills, and the consolidation of the theoretical knowledge studied [6,8,11,12].

The model of higher education in health sciences—and more specifically in nursing—needs training that reflects the workplace reality in which students will find themselves when they finish their studies [4]. A literature review identified few study that focuses on nursing students. Students have expressed that a more focused model needs to be implemented for the specific context of provision of healthcare. The teaching round in nursing education has been little implemented and studied. Considering all the above, this study’s objective is to determine whether nursing students’ clinical teaching carried out through the teaching round (with the model centered in the real world) is associated with a better acquisition of knowledge.

## 2. Methods

A controlled experimental, prospective study was conducted in April and May 2019, with a randomized allocation to two groups. This corresponds with the time period during which the students have their clinical practical placements, Practicum I, in the healthcare centers.

### 2.1. Population Selection

The reference population was second-year students of a nursing degree from the faculty of Health Sciences at the University of Jaén (Spain). The inclusion criteria were as follows: The participant must be older than 18 years and have agreed to participate. Exclusion criteria were as follows: those undertaking practical placement in a service that treats patients with pathologies whose theory has not yet been studied in class, such as geriatrics and the neonatal unit, and those who have previous experience in clinical placement during the nursing degree.

This study was approved by the ethics committee of the University of Jaén with the reference code FEB19/6PRY. All participants signed written informed consent, in accordance with the principals of the most recent Declaration of Helsinki, WMA General Assembly Brazil, 2013.

**Figure 1:**
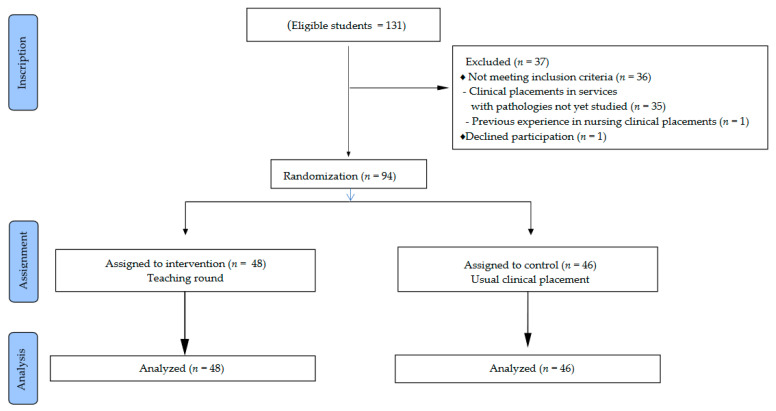
Flow diagram of the students.

The sample size necessary to detect that 40% of the students in the control group and 70% in the teaching round group have a score on the knowledge test greater than 8 points, with a power of 80% and an alpha risk of 0.05, is 42 subjects per group. As the population was small (131 subjects), it was decided that we include the whole population. After applying the inclusion and exclusion criteria, 94 students were eligible for inclusion (Figure 1).

### 2.2. Randomization

Students were randomly allocated to either the control or intervention group using a computer-generated random numbers table created with the software Epidat 4.1. The sequence was kept in individual opaque envelopes that were opened when a student met the inclusion criteria.

### 2.3. Intervention

#### 2.3.1. Teaching Round Group

A professional responsible for tutoring the students in the healthcare center conducted visits to the service (evaluating different patients who had been admitted) with a small group of students (5–6). During these sessions, they addressed the different subjects that had been covered during the theoretical classes, workshops, laboratory practicals, etc. Content is proposed in the teaching guides for the different subjects. Prior to the teaching round, the responsible professionals selected the patients they would evaluate in terms of pathology so that each case selected could be considered to support what had been seen in class or in practical placements. Various subjects were selected with differing needs, pathologies, symptoms, care requirements, etc., but always those that had already been covered in class. Some of the topics that were addressed included care and techniques in the surgical patient, care in the patient with respiratory disease (e.g., COPD, respiratory infections), and care in the patient with heart disease (e.g., acute myocardial infarction, respiratory failure).

#### 2.3.2. Control Group

The control group undertook practical placements according to the traditional model used in the faculty. In this model, the students attend the service where they do their placement and are tutored by a nurse. The students undertake the tasks that their reference nurse indicates during their time on the wards. These students also addressed the same issues as those in the intervention group.

### 2.4. Study Variables

The dependent variable was participation in teaching rounds. The principal effect was the acquisition of knowledge by the students during their practical placement.

In addition, data were collected on the sociodemographic variables of the student, their parents, academic information, health, motivation for choosing nursing, satisfaction with the intervention, and variables related to career development, which in this study entailed motivation, expectations, and evaluation of the nursing degree.

### 2.5. Data Collection

Data were collected using a questionnaire we designed that was filled out by the student. The questionnaire included questions about personal data such as date of birth, work, medical history, disability, place of residence during the course, level of study of the student and parents, income level of the student and parents, and average grade. Questions also included content regarding nursing career expectations, reasons for choosing nursing, satisfaction with the development of their studies, and motivation. The questionnaire had been previously piloted. In addition, to verify knowledge acquisition, students took a multiple-choice test on the contents that the students (in both groups) had seen in theoretical class and during their clinical placements. A register was created as a record for the students who agreed to participate in the study.

On finishing the practical placement (Practicum I), all students included in the study took a knowledge test on the material that they had seen during the day-to-day of the clinical placements to assess the acquisition of knowledge during these placements. The grade received during the course Practicum I, which corresponds to the students’ clinical placements in the healthcare centers, was also collected.

### 2.6. Data Analysis

First, we verified that randomization had worked. For the continuous effect variables, a t-test was used, whereas for categorical variables, the Fisher exact test was applied. If there were imbalances between the experimental and control groups, these variables were adjusted for in the multivariate analysis. In the multivariate analysis, we also adjusted for the student’s academic record, the presence of illness, and sex of the student. In the case of continuous effect variables, the analysis of covariance was applied; for categorical effect variables, logistic regression was used, and for ordinal effect variables, a polytomous logistic regression was used.

For all analyses, a value of α = 0.05 was considered as significant. Statistical analysis was performed using Stata (15.0, StataCorp L.P., College Station, TX, USA).

## 3. Results

The intervention group included 48 students who participated in the teaching rounds. The control group included 46 students who completed their practical placements as usual.

The two groups are compared in Table 1 in terms of different sociodemographic, academic, and health variables. No significant differences were found for age, education level, or previous illnesses between the two groups. The intervention group students had a mean age of 22.67 ± 6.96 years compared with 21.76 ± 5.47 years in the control group (*p* = 0.486). The intervention group was 82.61% (38) females compared to 89.58% (43) in the control group (*p* = 0.486). During the course, 52.08% (25) students in the intervention group lived in a shared flat with flatmates, while in the control group, 52.17% (24) lived in a shared flat (*p* = 0.644). In the intervention group, 10.42% (5) of the students were employed during the course, compared with 8.70% (4) in the control group (*p* = 0.527). The mean grade out of a maximum of 10 in the intervention group was 7.55 ± 0.72 versus 7.43 ± 0.58 in the control group (*p* = 0.373).

In terms of the students expressing whether or not they liked the degree, in the intervention group 95.83% (46) of the students responded “yes” compared with 97.83% (45) in the control group (*p* = 0.516). In the intervention group, 91.67% (44) felt that the nursing degree met their expectations compared with 97.83% (45) of the control group (*p* = 0.194). Both groups coincided in their belief that nursing was the career they had always wanted to do, which is the reason that led them to opt for this degree (Table 2).

In Table 3, the association between participation in the teaching round and the knowledge test is shown. The intervention group achieved a mean grade of 8.83 ± 0.22, and the control group had a mean grade of 7.68 ± 0.23 out of a maximum of 10 (*p* = 0.001). Table 3 also shows the Practicum I course evaluation, with a mean grade in the intervention group of 8.92 ± 0.09 versus 8.77 ± 0.09 in the control group (*p* = 0.279).

**Figure 2:**
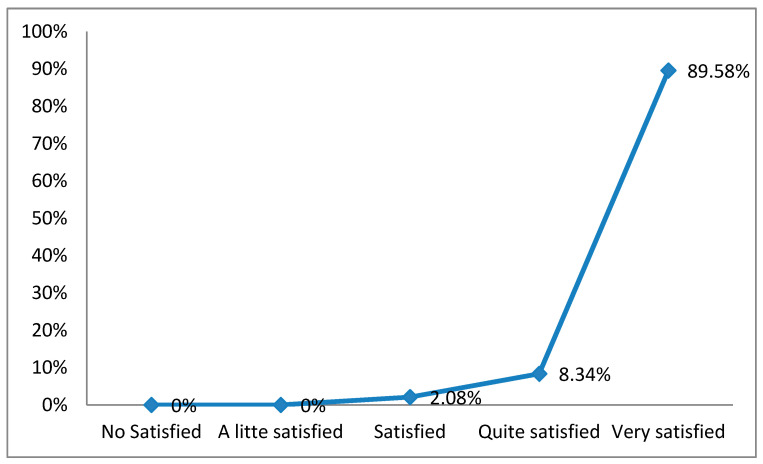
Satisfaction of the participating student in the intervention (teaching round).

The satisfaction among the students that participated in the teaching round was high: 89.58% (43) were very satisfied with the development of this intervention. Only 8.34% (4) were quite satisfied, and 2.08% (1) felt satisfied. No student indicated that they were not at all satisfied or only a little satisfied (Figure 2).

## 4. Discussion

Those students who participated in the teaching round obtained a higher grade in the knowledge test. Their grade was also higher for their global evaluation of the Practicum I course; however, this was not statistically significant.

Hidalgo Blanco, in an observational study of 378 nursing students at the University of Barcelona in Spain, found that after students completed their first year of a nursing degree, they felt their expectations had not been met; this contrasts with what we observed in our study, in which the majority of the students felt assured that their expectations were fulfilled [13]. This difference may be because in the first year of the degree in Hidalgo Blanco’s study, the subjects studied are more general, such as physiology or anatomy. However, in our study, which was carried out in the second year of the nursing degree, the subjects are more specific to the nursing field, such as clinical nursing or pediatric nursing. Even in the second year, students have already had practical placements in both the laboratory and in health centers. Therefore, it is possible that studying content that is focused on nursing better meets the expectations of students who chose to study nursing.

In line with our results, Zhang (2015) found in an experimental study, conducted in Shanghai with 120 nursing students who participated in teaching rounds and another 120 students in the control group who had practical placements as per the traditional model, that teaching rounds increased students’ knowledge, which in Zhang’s study was focused on nosocomial infections [14]. The results of a study conducted in Cuba, albeit in medicine, also demonstrated that the teaching round allows the student to develop clinical skills using immediate feedback with critique and self-critique, and that it fosters the development of their own criteria [15]. Other authors have also made conclusions along the same lines [16,17].

Our students were satisfied with this new teaching method, and our findings are comparable to those from Staun et al. (2010), who used a similar methodology [18]. Students, for the most part, as shown in literature, are satisfied with the implementation of new teaching methodologies; this point was demonstrated by Lipsky et al. (2019), who found high scores when assessing student satisfaction with a new model implemented in several US campuses [19]. Rahnavard et al. (2013) in an experimental study with 104 nursing students evaluating a new teaching method, also identified high student satisfaction with this new methodology [20]. In general, nursing students are quite satisfied with the training carried out in the clinical environment, as found and demonstrated by D’Souza et al. in a study with 380 undergraduate nursing students in Oman [21]. Nursing students accept well the introduction of new methods and tools in their learning process [22].

We did not find any influence of the teaching round on the overall grade of the course (Practicum I), although it must be kept in mind that the overall grade is the sum of multiple components outside the acquisition of skills and knowledge, such as punctuality, appropriate dress, among others.

Among the study’s limitations is the sample size; despite including a large part of the population, the sample size was still small. Among the strengths, it should be noted that randomization worked properly, as no significant differences were found between both groups. Therefore, any differences between both groups of women should not be due to chance. The collection of information was done by the students’ teachers, which may introduce a classification bias as the students may want to respond with answers that they believe will please the teachers. However, this bias was minimized as information was gathered through an anonymous heteroadministered questionnaire. The questions were formulated in a clear and understandable manner so that information bias can be ruled out. A selection bias associated with the nonresponse is unlikely to have had an influence on the results, as the response of the students to the recruitment was very positive with only one refusal to participate, and nothing suggests that the one who did not respond would have done so differently from those that did.

## 5. Conclusions

The teaching round increased the student’s acquisition of knowledge, although this was not reflected in the global grade of the course. Overall, the teaching round helped students to consolidate knowledge, and students who participated in the teaching round appear quite satisfied with the intervention. Therefore, we propose the implementation of this clinical practice model in nursing education to increase the quality of education and close the gap between nursing education and real-world clinical care.

## Figures and Tables

**Table 1:** Characteristics of the study sample.

Variable	Intervention Group *n* = 48	Usual Practical Placement *n* = 46	*p* Value
Age, M ± SD	22.67 ± 6.96	21.76 ± 5.47	0.486
Sex, *n* (%)			0.381
Male	5 (10.42)	8 (17.39)	
Female	43 (89.58)	38 (82.61)	
Civil status, *n* (%)			0.762
Single	40 (83.33)	36 (78.26)	
Married	2 (4.17)	2 (4.35)	
De facto relationship	5 (10.42)	8 (17.39)	
Divorced	1 (2.08)	0 (0)	
Education level, *n* (%)			0.697
Baccalaureate	34 (70.83)	29 (63.04)	
Vocational training	10 (20.84)	11 (23.92)	
University	4 (8.33)	6 (13.04)	
Paternal Education level, *n* (%)			0.333
No education	4 (8.33)	2 (4.35)	
Primary	5 (10.42)	5 (10.87)	
Incomplete secondary education, *n* (%)	5 (10.42)	4 (8.70)	
Secondary, *n* (%)	7 (14.58)	14 (30.43)	
Baccalaureate	6 (12.50)	6 (13.04)	
Vocational training	8 (16.67)	10 (21.74)	
University	13 (27.08)	5 (10.87)	
Maternal Education level, *n* (%)			0.118
No education	2 (4.17)	1 (2.17)	
Primary	3 (6.25)	4 (8.70)	
Incomplete secondary education, *n* (%)	6 (12.50)	1 (2.17)	
Secondary, *n* (%)	7 (14.58)	17 (36.96)	
Baccalaureate	10 (20.83)	9 (19.57)	
Vocational training	6 (12.50)	6 (13.04)	
University	14 (29.17)	8 (17.39)	
Parents’ civil status, *n* (%)			0.552
Married	42 (87.51)	44 (95.65)	
De facto relationship	1 (2.08)	0 (0)	
Divorced	4 (8.33)	2 (4.35)	
Separated	1 (2.08)	0 (0)	
Income level, *n* (%)			0.971
<1000 Euros/month	9 (18.75)	8 (17.39)	
1000 1999 Euros/month	21 (43.75)	21 (45.65)	
2000 2999 Euros/month	12 (25.00)	10 (21.74)	
≥3000 Euros/month	6 (12.50)	7 (15.22)	
Living situation during the academic year, *n* (%)			0.644
Family home	21 (43.75)	22 (47.83)	
Student residence	2 (4.17)	0 (0)	
Shared flat	25 (52.08)	24 (52.17)	
Employment during the course, *n* (%)			0.527
No	43 (89.58)	42 (91.30)	
Yes	5 (10.42)	4 (8.70)	
Presence of an illness, *n* (%)			0.090
No	41 (85.42)	44 (95.65)	
Yes	7 (14.58)	2 (4.35)	
Officially recognized disability, *n* (%)			0.292
No	47 (97.92)	43 (93.48)	
Yes	1 (2.08)	3 (6.52)	
Academic grade, M ± SD	7.55 ± 0.72	7.43 ± 0.58	0.373

Abbreviations: M = mean; SD = standard deviation.

**Table 2:** Association of variables related to motivation and career development.

Variable	Intervention Group *n* = 48	Usual Practical Placement *n* = 46	*p* Value
Student likes the profession, *n* (%)			0.516
No	2 (4.17)	1 (2.17)	
Yes	46 (95.83)	45 (97.83)	
The degree meets the student’s initial expectations, *n* (%)			0.194
No	4 (8.33)	1 (2.17)	
Yes	44 (91.67)	45 (97.83)	
The student feels motivated by the degree, *n* (%)			0.480
No	2 (4.17)	3 (6.52)	
Yes	46 (95.48)	43 (93.48)	
Reason for degree choice, *n* (%)			0.955
Did not have the required grade for the desired one	5 (10.42)	5 (10.87)	
Always wanted to study nursing	35 (72.92)	35 (76.09)	
For professional career options	3 (6.25)	3 (6.52)	
Family tradition	5 (10.42)	3 (6.52)	
Others	0	0	

**Table 3:** Effect of the intervention (teaching ward round) in the acquisition of knowledge and practical clinical grades.

Variable	Crude Analysis			Multivariate Analysis *		
	Intervention Group M (SEM)	Usual Practical Placement M (SEM)	*p* Value	Intervention Group M (SEM)	Usual Practical Placement M (SEM)	*p* Value
Grade in knowledge test (maximum 10)	8.83 (0.17)	7.67 (0.26)	˂0.001	8.83 (0.22)	7.68 (0.23)	**0.001**
Grade in practical placement I (maximum 10)	8.94 (0.08)	8.76 (0.10)	0.169	8.92 (0.09)	8.77 (0.09)	0.279

* Adjusted for sex, illness, and academic grade record; Abbreviations: M = mean; SEM = standard error of mean; Bold: statistically significant differences.

## Data Availability

The datasets used and/or analysed during the current study are available from the corresponding author on reasonable request.
